# Basic life support knowledge transfer from schoolchildren to family members and friends: a scoping review and preliminary conceptual framework for training implementation

**DOI:** 10.1016/j.resplu.2026.101407

**Published:** 2026-07-10

**Authors:** María García-Martínez, Santiago Martínez-Isasi, María Sobrido-Prieto, Luis Castro-Alonso, Antonio Rodríguez-Núñez

**Affiliations:** a Faculty of Nursing, University of Santiago de Compostela, Santiago de Compostela, Spain; b CLINURSID Research Group, University of Santiago de Compostela, Santiago de Compostela, Spain; c Simulation, Life Support and Intensive Care Research Unit of Santiago de Compostela (SICRUS). Simulation and Intensive Care Research Unit of Santiago (SICRUS), Health Research Institute of Santiago de Compostela (IDIS), Departamento de Psiquiatría, Radiología, Salud Pública, Enfermería y Medicina, University of Santiago de Compostela, Santiago de Compostela, Spain; d Faculty of Health Sciences, University of A Coruña (UDC), A Coruña, Spain; e Pontevedra Public Hospital, Galician Public Health System, Pontevedra, Spain; f Pediatric Critical, Intermediate and Palliative Care Section. University Hospital of Santiago de Compostela, Galician Public Health System, Santiago de Compostela, Spain; g Spanish Network in Maternal, Neonatal, Child and Developmental Health Research (RICORS-SAMID, RD24/0013/0023), Instituto de Salud Carlos III, 28040 Madrid, Spain

**Keywords:** Basic life support, Cardiopulmonary resuscitation, Heart arrest, Adolescent, Health education, Family, Bystander

## Abstract

**Introduction:**

Out-of-hospital cardiac arrest is a leading cause of death in Europe, with most events occurring at home. Early recognition and initiation of resuscitation improve survival and neurological outcomes, yet they are not consistently performed by bystanders. School-based initiatives such as “Kids Save Lives” promote basic life support (BLS) training and encourage schoolchildren to disseminate this knowledge within their families. However, evidence on how this transfer occurs and how it can be assessed remains limited.

**Aim:**

To map how BLS knowledge transfer from schoolchildren to family members and friends has been implemented and evaluated, and to identify potential key limitations for measurement and reporting.

**Methods:**

A scoping review was conducted following PRISMA-ScR. Searches were performed in PubMed, Scopus, and Web of Science in February 2026. Studies involving adolescents (10–19 years) trained in BLS at school who subsequently taught family members and friends were included. Evidence was synthetized descriptively, focusing on training characteristics, transfer processes, and reported outcomes.

**Results:**

Four quasi-experimental studies and one prospective observational study were included. Training was mainly based on self-instruction kits, and knowledge transfer was typically conducted without supervision. Outcomes focused primarily on theoretical knowledge, with limited assessment of practical skills or long-term retention. The reported multiplier effect ranged from 0.33 to 4.9, with substantial variability and inconsistent measurement.

**Conclusion:**

Schoolchildren may act as transmitters of BLS knowledge to family members and friends, but current evidence is limited, heterogeneous, and largely based on indirect or self-reported outcomes. More structured and standardized approaches are needed to better understand, evaluate, and optimize this potentially relevant knowledge transfer process.

## Highlights

• Adolescents may act as multipliers of BLS knowledge in the community.
• Evidence on family-based CPR knowledge transfer remains scarce.
• Current studies rarely assess practical skills or long-term retention.
• Transfer interventions are highly heterogeneous and poorly standardized.
• A conceptual framework is proposed to guide future implementation and evaluation.

## Graphical abstract

**Figure:**
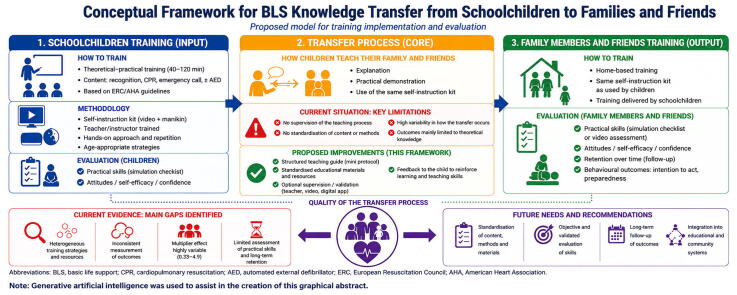

## Introduction

Out-of-hospital cardiac arrest (OHCA) remains one of the leading causes of death in Europe, with the majority of events occurring in the home setting.^1^, ^2^ Early recognition and prompt initiation of cardiopulmonary resuscitation (CPR) by bystanders, together with timely defibrillation, are key determinants of survival and neurological outcome.^3^, ^4^

Nevertheless, the bystander CPR rate may range from 13% to 82% (average: 58%) between European countries.^1^ In this context, one of the strategies with the greatest international consensus to increase bystander CPR is the teaching of CPR in schools.

Since 2015, the European Resuscitation Council (ERC) has launched the “Kids Save Lives” initiative, which promotes the teaching of CPR in schools.^5^ The scientific literature supports the feasibility and effectiveness of school-based CPR training, describing a variety of pedagogical strategies, methodologies, and teaching resources tailored to students’ developmental stages.^5^, ^6^

Previous systematic reviews have examined which first aid and CPR skills are appropriate for schoolchildren according to their developmental stage.^7^, ^8^ These studies support the feasibility and effectiveness of school-based CPR education. However, they primarily focus on skill acquisition among students themselves and do not address the potential secondary dissemination of knowledge beyond the classroom. Therefore, in addition to training and equipping students with life-saving skills, the “Systems Save Lives”, chapter of the ERC 2025 Guidelines, proposes that schoolchildren might act as multipliers of knowledge within their households, suggesting that a single trained student could potentially train up to ten relatives (potential multiplier effect).^6^, ^9^, ^10^, ^11^, ^12^

This assumption, however, has been largely programmatic and its empirical basis remains unclear. The teaching strategy, methodology, and resources employed for this purpose have not been defined, and, to our knowledge, no conceptual framework for implementation currently exists, although similar mechanisms have been observed in other public health domains, where children influence household behaviors.^12^

Despite policy recommendations encouraging students to disseminate CPR skills within their households, it remains unclear how such knowledge transfer occurs, how it has been implemented, and how it has been evaluated. Therefore, we conducted a scoping review to systematically map existing evidence on the transfer of basic life support (BLS) knowledge, attitudes, and skills from adolescents to their family members and friends, and to identify key gaps in research and implementation.

## Methods

The review followed the methodological framework for scoping reviews as described by Arksey and O’Malley^13^ and reported according to PRISMA-ScR.^14^ It was registered in Open Science Framework (OSF) (available at https://doi.org/10.17605/OSF.IO/PG42E).^15^ The research question was: How has the transfer of BLS knowledge, skills and attitudes from schoolchildren to family members and friends been implemented and evaluated?

### Eligibility criteria

Studies were included according to the PCC framework. Population: adolescents aged 10–19 years, as defined by the World Health Organization,^16^ who had received school-based BLS training. Concept: transfer of BLS knowledge, skills and attitudes from school-trained adolescents to family members and/or friends through educational activities delivered by the adolescents. Context: school-based BLS educational programs incorporating a subsequent transfer component outside the school setting.

Study characteristics: experimental, quasi-experimental and observational primary studies published in English or Spanish between 2011 and 2025 were considered. This period was selected to include the five years preceding the 2015 Kids Save Lives recommendation and all subsequent literature potentially influenced by this initiative. Purely descriptive studies not examining a transfer intervention were excluded**.**

### Search: Information sources and Search strategy

A comprehensive search was conducted in PubMed, Scopus, and Web of Science in February 2026 (see Supplement). The results were exported to the reference manager Zotero to remove duplicates. Since reference managers do not detect 100% of duplicates, a manual review was conducted by comparing titles, authors, and publication years. In addition, one eligible study was identified through supplementary manual searching and incorporated into the review. In addition, backward citation searching of all included studies was systematically performed to identify potentially relevant records.

### Study selection

**Fig. 1:**
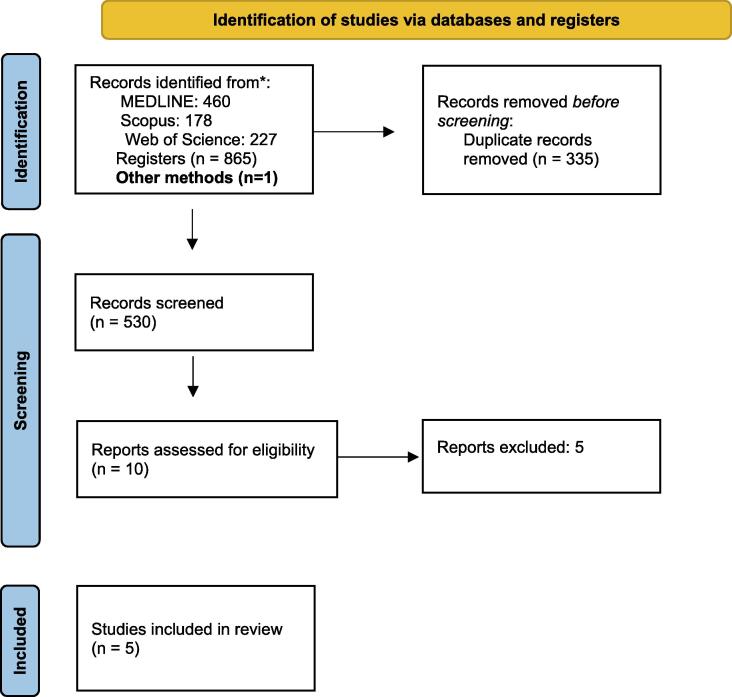
**PRISMA-ScR flowchart of identification and selection of studies**.

Title and abstract screening, as well as full-text eligibility assessment, were conducted independently by two reviewers (MGM and MSP) using a Microsoft Excel spreadsheet specifically developed for study selection and eligibility assessment. Disagreements were resolved by discussion and, when necessary, consultation with a third reviewer (SMI) (Fig. 1).

### Data charting

A standardized data extraction form was developed. Two reviewers (MGM and LCA) independently extracted data using a predefined template in April 2026. Discrepancies were resolved by discussion and, when necessary, with the involvement of a third reviewer (SMI). Data were extracted on study characteristics, training interventions, and knowledge transfer to family members and friends. For the training of adolescents, variables included sample, training method, duration, materials, and outcomes (knowledge and retention). For knowledge transfer to family members and friends, the same variables were collected, including outcome assessment. Given the two levels of analysis, data were organized into two tables: (1) training of adolescents and (2) knowledge transfer to family members and friends. All studies reported a multiplier effect, defined as the ratio between the number of individuals ultimately reached (directly or indirectly) and those initially trained.

### Critical appraisal

We undertook methodological appraisal to characterize the nature and methodological features of the available evidence. The appraisal was performed independently by two reviewers (MGM and MSP) using the JBI Critical Appraisal Checklist for quasi-experimental studies.^17^ This process did not inform study exclusion, weighting or interpretation of results (Appendix 2).

### Synthesis of results

Results were synthesized descriptively through tabulation and a narrative approach. Data were organized into two domains: (1) training of adolescents and (2) knowledge transfer to family members and friends. Within each domain, training characteristics, transfer processes, outcome measures (knowledge, attitudes, retention), and reported effects, including the multiplier effect, were summarized using tables and narrative synthesis to identify patterns, heterogeneity, and gaps in the evidence.

## Results

The initial search identified 530 records. After screening, four studies met the inclusion criteria and were analyzed.^18^, ^19^, ^20^, ^21^ In addition, one study was included by backward citation searching^22^ (Fig. 1). Across studies, training was mainly based on self-instruction kits and was delivered without supervision during the transfer process. Outcome assessment focused primarily on theoretical knowledge, with limited evaluation of practical skills or long-term retention. The multiplier effect was inconsistently defined and measured across studies.

### Study characteristics

Four studies were quasi-experimental and one was a prospective observational study. All the studies had a non-randomized, convenience sample.^18^, ^19^, ^20^, ^21^, ^22^ Most studies met more than 80% of JBI items.^17^ Four of the studies were conducted in Europe (Italy,^18^ Belgium,^20^ Poland^21^ and Spain^22^ and one in the United States^19^).

### Participants characteristics

A total of 1566 students were included, with sample sizes ranging from 71^19^ to 600^18^ (Table 1); all were adolescents aged 10–19 years. In two studies,^20^, ^21^ fewer than 26% of schoolchildren had prior first aid education, while this information was not specified in the remaining studies.^19^, ^22^ The sample size of relatives ranged from 1.136^22^ to 347.^19^ This information was not reported in one study.^18^

**Table 1:** General characteristics of studies.

**Study**	**Country**	**Design**	**JBI**	**Sample size**	**Prior training**
Corrado et al.^18^	Italy	Quasi-experimental (pre-post)	6.5	Adolescents: 600 Relatives and friends: Not specified	17.3%
Ríos et al.^19^	Chicago (EEUU)	Quasi-experimental (pre-post)	7	Adolescents: 71 Friends and family members: 347	−
Stroobants et al.^20^	Belgium	Quasi-experimental (pre-post)	6	Adolescents: 290 Relatives and friends: 874	25.1%
Iskrzycki et al.^21^	Poland	Quasi-experimental (pre-post)	6	Adolescents: 133 Parents: 361	−
Pardo- Ríos et al.^22^	Spain	Prospective observational	7	Adolescents: 472 Family members/close contact: 1.136	−

### Training characteristics

Guidelines Used (Table 2): AHA guidelines were reported in two studies,^18^, ^19^ ERC guidelines in three studies^18^, ^20^, ^22^ and one study did not specify the guideline followed.^21^

**Table 2:** Training adolescents.

Study	**Corrado et al.**^18^	**Rios et al.**^19^	**Stroobants et al.**^20^	**Iskrzycki et al.**^21^	**Pardo-Ríos et al.**^22^
Guidelines	AHA 2005/ERC	AHA	ERC	NS	ERC 2025
Age of the participants	16.5 ± 0.8 years old	14–16 years old	11–13 years old	16–19 years old	10–12 years old
BLS	Unresponsive Breathing Call Emergencies	Unresponsive Breathing	Unresponsive Breathing Call Emergencies	Safety place Unresponsive Breathing	Safety place Unresponsive Breathing Call Emergencies
CPR	Chest compressions Breathing	Chest compressions	Chest compressions	Chest compressions Breathing	Chest compressions
AED	Included in the video but not shown	Trained but not evaluated	NS	NS	Trained but not evaluated
Methods	Theory + Practice	Theory + Practice	Theory + Practice	Theory + Practice	Theory + Practice
Resources	Autokit: DVD (27′) + MiniAnne Laerdal	Autokit:Video+ Manikin CPRAnytime	Autokit: Video (30′)+ Manikin Minipop	NS	Low-cost Manikin+ Presentation
Sessions	One session: 67′ Theory: 27′ (video) Practice:40′	Two sessions: 45′ each one Theory: NS Practice: NS	NS	One session: 120′ Theory: NS Practice: NS	One session: 60′ Theory: 30′ Practice: 30′
Instructors	Physical education teachers + Cardiologists Como Volunteers − Cuore	Teacher + CPR Instructor (emergency physicians) if needed	Previously trained teachers	NS	School nurses who (trained by CPR Instructors)
Evaluation method	Questionnaire	Questionnaire	Questionnaire (Web)	Questionnaire	Questionnaire (Checklist)
Time of evaluation	8 months after training	Pre-training Immediately after	Immediately after	Pre-training Immediately after 4 months after training	None
Attitudes	Self-reliance Self-confidence	NS	Intention (willingness to)	Attitudes towards CPR (no further details are given)	None

AHA: American Heart Association; ERC: European Resuscitation Council; BLS: Basic Life Support; CPR: Cardiopulmonary Resuscitation; AED: semi-automatic defibrillator; NS: not specified.

Content Delivered and Type of Training: All studies covered the BLS algorithm and chest compressions.^18^, ^19^, ^20^, ^21^, ^22^ Two studies also included CPR with ventilations^18^, ^21^ and the use of AED was specifically addressed in two studies.^19^, ^22^

Adolescents: Training sessions were theoretical-practical and lasted between 40^18^ and 120 min.^21^ Three studies^18^, ^19^, ^20^ used an auto-kit consisting of a video and manikin, and one study reported the use of a low-cost auto-kit.^22^ Iskrzycki et al.^21^ did not specify the type of teaching material used.

Instructors included cardiologists,^18^ previously trained teachers,^18^, ^19^, ^20^ program volunteers,^18^ school nurses,^22^ emergency physicians.^19^ Iskrzycki et al.^21^ did not specify this point.

Relatives: In four studies^18^, ^19^, ^20^, ^22^ the relatives were trained by adolescents using the same auto-kit with which the students had previously trained. Iskrzycki et al.^21^ did not specify this point.

### Transfer of knowledge from schoolchildren to family members and friends

Four studies used the same training methodology: the schoolchildren themselves, previously trained, took on the role of peer instructors and transmitted the knowledge acquired during their training using an auto-kit, the same one with which their training was previously conducted.^18^, ^19^, ^20^, ^22^ This was not specified in one study.^21^

Assessment of family members and friends was mainly based on written questionnaires, with variations in timing and administration across studies.^19^, ^20^, ^21^ In one study, knowledge was not assessed,^18^ while others used pre-post or post-training questionnaires, including web-based formats and follow-up at up to four months.^19^, ^20^, ^21^ In one study, parental training was assessed using a home video.^22^

The reported multiplier effect ranged from 0.33 to 4.9 across studies^19^, ^21^, ^22^ (Table 3), showing substantial variability between interventions. This multiplier effect reflects the number of additional individuals who received BLS training through knowledge transfer from each trained schoolchild, highlighting the potential of school-based programs to extend CPR education beyond the classroom and into the community.

**Table 3:** Knowledge transfer to family members and friends by adolescents previously trained in BLS.

Study	**Corrado et al.**^18^	**Rios et al.**^19^	**Stroobants et al.**^20^	**Iskrzycki et al.**^21^	**Pardo-Ríos et al.**^22^
Guidelines	AHA 2005/ERC	AHA	ERC	NS	ERC 2025
Age of the participants	NS	NS	NS	Families 38–59 years old	NS
BLS	NS	NS	NS	NS	Safety place Unresponsive Breathing Call Emergencies
CPR	NS	NS	NS	NS	Chest Compressions
AED	NS	NS	NS	NS	NS
Methods	NS	NS	NS	NS	NS
Resources	Autokit:DVD (27′) + MiniAnne Laerdal	Autokit: Video + Manikin: CPRAnytime	Autokit: Video (30′) + Manikin: Minipop	NS	Low-Cost Manikin
Sessions	NS	NS	NS	NS	NS
Evaluation method	None	Questionnaire	Questionnaire (web)	Questionnaire	Home video
Attitudes	NS	NS	Intention (willingness to)	NS	NS
Multiplier effect*	1.77	4.9	1.70	0.33	2.41

AHA: American Heart Association; ERC: European Resuscitation Council; BLS: Basic Life Support; CPR: Cardiopulmonary Resuscitation; AED: semi-automatic defibrillator; NS: not specified.

* Multiplier effect: proxy indicator of the impact on family members generated by the students' intervention.

### Proposed conceptual framework of knowledge transfer

Based on these findings, a preliminary conceptual framework was developed to represent the process of knowledge transfer from schoolchildren to family members and friends. It integrates key components identified across studies, including training characteristics, transfer mechanisms, and reported outcomes. This framework offers an initial representation of a process that has been widely assumed but not explicitly modeled and offers a basis to move from assuming knowledge transfer to explicitly designing and evaluating it.

We structured the framework into three interrelated domains: (1) characteristics of the initial training intervention, (2) the transfer process from schoolchildren to family members and friends, and (3) transfer outcomes.

#### Training characteristics

Includes factors such as training format (e.g., self-instruction kits or instructor-led sessions), duration, and level of supervision. These elements may condition the child’s understanding, confidence, and readiness to transmit knowledge, as well as the consistency of the transfer process.

#### Transfer process

Describes how children communicate and demonstrate BLS knowledge to family members and friends through verbal explanation, practical demonstration, or a combination of both. This stage is particularly relevant because it influences how effectively knowledge and skills are transferred beyond the school setting. Factors such as the child’s active engagement, communication abilities, and the presence or absence of supervision may influence the accuracy and quality of knowledge transmission

#### Outcomes

Includes effects on family members, such as knowledge acquisition, attitudes towards BLS, and willingness to perform resuscitation. Current outcome assessment is limited and does not fully capture the effectiveness of knowledge transfer, as most studies rely on self-reported measures, with limited assessment of practical skills or long-term retention.

We consider this framework highlights the complexity of knowledge transfer and may provide a structured basis for future research and more standardized training and evaluation strategies. It could also help identify key intervention points, particularly the need to support the transfer process and to include objective, standardized outcome measures.

## Discussion

This scoping review explored the available evidence on how BLS knowledge is transferred from schoolchildren to their family members and friends. Although school-based BLS training is increasingly promoted, the evidence specifically addressing this transfer process remains limited and heterogeneous. Currently, several questions remain unanswered: Current data do not allow determining whether adults acquire useful skills after this training or retain them over time. It is also unclear to what extent schoolchildren can reliably act as peer at-home instructors after brief training at school.

Our review found very limited evidence to address these relevant questions. The few and heterogeneous studies that have examined the impact of the transfer of BLS knowledge and skills from schoolchildren to family members and friends^18^, ^19^, ^20^, ^21^, ^22^ suggest that, although school-trained adolescents can train their relatives at home, there is still insufficient evidence regarding the level of skills and competencies acquired. Therefore, it is difficult to determine whether family members would be able to perform these maneuvers in a real-life situation, raising concerns about the true impact of these educational interventions. Furthermore, studies focus on the immediate environment of schoolchildren, which limits the generalizability of the findings beyond this context.^18^, ^19^, ^20^, ^21^, ^22^ Future studies should explore whether knowledge transfer differs across types of family members and friends, as factors such as age, cohabitation, and prior exposure to health information may influence both engagement and learning outcomes. In addition, it would be necessary to assess the transfer of practical skills, and not just knowledge, in order to evaluate the real impact of these interventions. Further research should focus on how this transfer occurs, under what conditions it is most effective, and how it translates into practical skills and retention over time.

The included studies involved adolescents taking simple training materials home and acting as peer instructors for their family members and friends, without expert supervision and after receiving only brief prior training at school, typically around two hours. Although this approach might offer advantages in terms of simplicity and accessibility, it raises concerns about the quality of learning achievable by untrained adults. The effectiveness of this process appears to depend on the interaction between capability (skills acquired during training), opportunity (family context and resources), and motivation (engagement and willingness), as described in behavior change frameworks.^23^ The absence of professional verification of learning not only limits the validity of results but also prevents a clear understanding of knowledge transfer and the actual capacity to respond to a cardiac arrest event. To improve the validity of training interventions targeting family members and friends, future studies should incorporate more structured instructional models, including supervision, feedback, or competency validation, ensuring the quality of the transfer process and securing a tangible impact on the chain of survival.

On the other hand, most studies rely on self-reported measures, which may overestimate effectiveness. Objective assessment of practical skills and long-term retention is limited. In addition, the “multiplier effect” is often assumed rather than properly evaluated, and its magnitude and reproducibility remain unclear.

Moreover, the absence of evaluation of attitudes or motivational variables leaves open the question of whether this methodology influences the willingness of family members and friends to act in the event of a cardiac arrest. This is a particularly relevant aspect, as readiness to intervene depends not only on knowledge but also on factors such as confidence, perceived self-efficacy, and motivation—areas that were not addressed in the reviewed studies.

Our scoping review has some limitations. The small number of studies and their heterogeneity limit the strength of the conclusions. Assessment of retention was limited and inconsistently reported, and few studies included follow-up beyond the immediate post-training period. This lack of longitudinal data represents an important limitation, as the retention of knowledge and skills is essential to ensure an effective response in real-life situations, which may occur months or even years after training. Nevertheless, the structured approach of this scoping review provides a clearer overview of the current state of the evidence and highlights relevant gaps. The proposed conceptual model is derived from a small and heterogeneous evidence and should be understood as exploratory rather than validated.

## Conclusions

The evidence suggests that schoolchildren may be able to transmit BLS knowledge to family members at home. Knowledge transfer should not be assumed as an automatic outcome of school-based training, but as a process that requires structured implementation and prospective assessment.

## Ethical statement

The scoping review was carried out using existing published data, with no identifiable information about individual patients. Consequently, approval from a research ethics committee was not deemed necessary.

## CRediT authorship contribution statement

**María García-Martínez:** Writing – original draft, Investigation, Data curation, Conceptualization. **Santiago Martínez-Isasi:** Project administration, Methodology, Investigation, Conceptualization. **María Sobrido-Prieto:** Writing – review & editing, Supervision, Resources, Methodology, Investigation. **Luis Castro-Alonso:** Writing – review & editing, Visualization, Methodology, Data curation. **Antonio Rodríguez-Núñez:** Writing – review & editing, Visualization, Validation, Supervision, Conceptualization.

## Funding

Research funded by Instituto de Salud Carlos III (ISCI-III)-PI23/00687 co funded by the European Union (EU).

Maria Garcia-Martinez is the recipient of a grant co-financed with funds from the University Staff Training Program (FPU2023) of the Ministry of Spain (File number: FPU23/03035).

## Declaration of competing interest

The authors declare that they have no known competing financial interests or personal relationships that could have appeared to influence the work reported in this paper.

Santiago Martínez Isasi served as a reviewer for Resuscitation Plus.

## Appendix A Supplementary material

Supplementary Data 1 The supplementary material includes a table listing the different search strategies used in the databases.
