# Supplementary material for: Basic life support knowledge transfer from schoolchildren to family members and friends: a scoping review and preliminary conceptual framework for training implementation
Source: Resusc Plus. 2026 Jul 10;30:101407. doi: 10.1016/j.resplu.2026.101407 (PMC13427584; doi:10.1016/j.resplu.2026.101407)
Supplement: Supplementary Data 1 — The supplementary material includes a table listing the different search strategies used in the databases. [file mmc1.docx]

**APPENDIX**

| Database | search strategy | Results |
| --- | --- | --- |
| PUBMED | ("Child"[Mesh] OR child* OR school* OR "Adolescent"[Mesh] OR adolescen* OR student*)  AND  ("Health Knowledge, Attitudes, Practice"[Mesh] OR "Health education" OR "education"[MeSH Terms] OR "education"[MeSH Subheading] OR "Teaching"[Mesh] OR teach* OR train* OR instruct* OR course OR learn* OR teach* OR Dissemin* OR implement* OR Transfer* OR “reverse training” OR "cascade training")  AND  ("Cardiopulmonary Resuscitation"[Mesh] OR "Cardiopulmonary Resuscitation"[TITLE] OR cpr[TITLE] OR bls [TITLE] OR "Basic Life Support"[TITLE])  AND  ("Social Networking"[Mesh] OR "Peer Group"[Mesh] OR "Friends"[Mesh] OR friend* OR Neighbor* OR relative* OR "Family" OR famil* OR father* OR parent* OR Mother* OR Brother* OR Sister*)  Filters: **English, Spanish portugy, from 2011 – 2025. Se excluirán editorials y notas.** | 460 |
| SCOPUS | (CHILD* OR school* OR adolescen* OR student*)  AND  (educa* OR teach* OR train* OR instruct* OR course* OR learn* OR Dissemin* OR implement* OR Transfer*)  AND  TITLE ("Cardiopulmonary Resuscitation" OR cpr OR bls OR "Basic Life Support")  AND  ("Peer Group" ORrelative* OR friend* OR neighbour* OR relative* OR famil* OR father* OR parent* OR mother* OR brother* OR sister*)  **English, Spanish, from 2011 – 2025. Se excluirán editorials y notas.** | 178 |
| WEB OF  SCIENCE | (CHILD* OR school* OR adolescen* OR student*)  AND  (educa* OR teach* OR train* OR instruct* OR course* OR learn* OR Dissemint* OR implement* OR Transfer*)  AND  TITLE ("Cardiopulmonary Resuscitation" OR cpr OR bls OR "Basic Life Support")  AND  ("Peer Group" OR relative* OR friend* OR neighbor* OR relative* OR famil* OR father* OR parent* OR mother* OR brother* OR sister*)  **English, Spanish, from 2011 – 2025. Se excluirán editorials y notas.** | 227 |
